# Molecular Dynamics Simulations Reveal a Broadly Open Solution Ensemble for the DNA Holliday Junction: Mg^2+^ Crossover Bridging and Weak Force-Field Sensitivity

**DOI:** 10.3390/ijms27219634

**Published:** 2026-10-28

**Authors:** Yonghui Zhang, Yujia Zhou

**Affiliations:** 1 Institute of Computing Science and Technology, Guangzhou University, Guangzhou 510006, China; 2 Huangpu Research School, Guangzhou University, Guangzhou 510006, China; 3 School of Biomedical Engineering, Southern Medical University, Guangzhou 510515, China; 4 Guangdong Provincial Key Laboratory of Medical Image Processing, Southern Medical University, Guangzhou 510515, China; 5 Guangdong Provincial Engineering Laboratory for Medical Imaging and Diagnostic Technology, Southern Medical University, Guangzhou 510515, China

**Keywords:** DNA Holliday junction, DNA nanotechnology, molecular dynamics, force-field assessment

## Abstract

Holliday junctions, four-way DNA crossovers central to DNA origami and key intermediates of homologous recombination, must be accurately modeled for rational DNA-nanodevice design, yet their solution conformational ensemble remains incompletely characterized. We characterize this ensemble for the experimental junction d(TCGGTACCGA)_4_ by all-atom molecular dynamics, comparing the AMBER force fields parmbsc1, OL21, and OL24 in OPC water with matched ionic conditions. In every force field, the junction explores a broad, nearly flat inter-arm-angle landscape, with <1 kcal/mol variation across inter-arm angles of 2–90°: the open state is favored by 0.45–0.63 kcal/mol, the crystallographic-like inter-arm angle occurs in 2–5% of frames, and the crossover remains coaxially stacked whether open or compact. A Mg^2+^ ion occupies the crossover pocket in 92–94% of frames, specifically bridging the A- and C-strand phosphates (Li–Merz CM Mg^2+^ set). Stacking-isomer exchange between the A/C-continuous and crystallographic B/D isomers proceeds at ~0.5 ns^−1^ in this minimal short-arm construct, independent of force field, with A/C-continuous favored ~2:1. parmbsc1 yields a more open junction than OL21 and OL24, which reproduce the crystallographic inter-arm angle of 39–43°; the sugar-pucker shift targeted in OL24 is also visible at the junction. These results establish a force-field-robust baseline for simulation-guided design of crossover-containing nanostructures.

## 1. Introduction

Holliday junctions (HJs) occupy a unique position at the interface of genome biology and structural nanotechnology. In vivo, they are the four-stranded intermediates of homologous recombination and DNA repair: two homologous duplexes exchange strands, the junction branch migrates, and structure-selective nucleases resolve it into crossover or non-crossover products [1,2]. Junction conformation is functionally read out: single-molecule studies resolved millisecond exchange between coaxially stacked stacking isomers [3,4,5], the tumor-suppressor protein BRCA1 prefers the open, planar form [6], and the identity of the continuously stacked strand pair controls how resolving enzymes approach the crossover [7]. In vitro, the same crossover motif serves as the elementary connection of DNA origami, where tens to hundreds of HJs hold designed nanostructures together [8,9]; the mechanical and conformational properties of each crossover directly determine the rigidity, dynamics and ultimately the function of the assembled device [10,11,12,13]. Beyond all-DNA constructs, the same design logic templates the assembly of nanoparticle arrays and DNA–inorganic hybrid materials with programmable optical, electronic and magnetic properties [14], with the crossover geometry setting the spacing and orientation of the attached functional groups. As DNA origami transitions from proof-of-concept structures toward functional devices—DNA cages, polyhedra and nanoscale containers that can encapsulate and release molecular payloads, or report on their chemical environment [15,16]—the accuracy with which simulations describe the HJ crossover becomes a practical bottleneck for the entire field.

Structurally, the HJ is among the best-characterized DNA motifs [17]. Förster resonance energy transfer (FRET) established the right-handed, antiparallel stacked-X as the canonical folded conformation in solution [18,19] and showed that low salt opens the junction into a planar-like ensemble [20]; crystallography captured the stacked-X geometry and its sequence- and ion-dependence for a family of inverted-repeat junctions [21,22,23]; and a consistent geometric vocabulary—interduplex angle, J-roll and J-slide—was defined by Watson et al. [24].

The Amber nucleic-acid force-field line—bsc0 → bsc1 → OL15 → OL21 → OL24—has progressed by correcting specific structural deficiencies [25,26,27,28,29]. The latest member, OL24 (2025), refines the sugar-pucker torsion potential (the five sugar torsions ν_0_–ν_4_) to correct the over-stabilization of A-form geometry [29]. Simulated junctions are sensitive to this choice, a sensitivity documented since the earliest junction simulations [30]: Zhang et al. quantified opening–closing free energies of a junction (J1) with OL15 and reported that AMBER force fields over-stabilize the closed/stacked state by ≈5–6 kcal/mol [31]; a concurrent preprint from the OL24 developer group examined HJ topology potentials of mean force (PMFs) under OL21/OL24 and found the parallel state metastable (<1% population) against the antiparallel global minimum [32]; and Singh et al. simulated the same d(TCGGTACCGA) junction studied here, reporting site-selective ion binding [33]. What is missing is a controlled, multi-force-field baseline: the parmbsc1 reference, the interduplex-angle (IDA) coordinate anchored to crystallographic values, ion behavior at the crossover, and an explicit sampling-error analysis; the most recent systematic force-field assessment is that of Love et al. [34]. Methodological reviews of in silico nucleic-acid modeling and recent molecular-dynamics studies of DNA complexes with peptides and other partners illustrate how broadly this toolkit now reaches [35,36,37].

**Figure 1:**
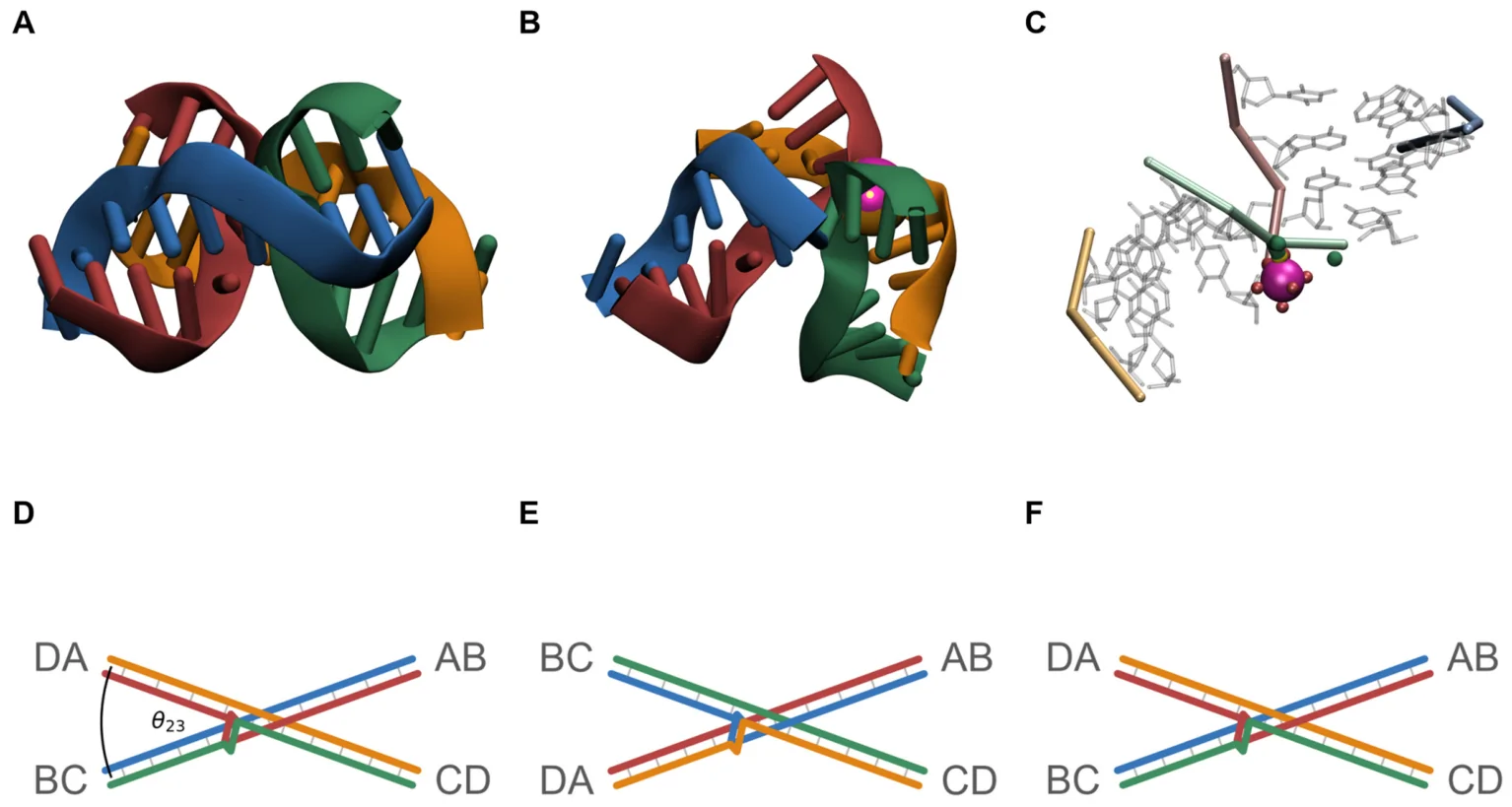
Structure renders and schematics of the simulated system, drawn with VMD [38]. (**A**) Crystallographic conformation of the d(TCGGTACCGA) junction (PDB 1M6G [22]); backbone ribbons and base plates coloured by strand (A red, B blue, C green, D orange). (**B**) A representative 200 ns solution frame (parmbsc1, replica 3), superposed onto the crystal coordinates: the junction is broadly open, and the single Mg^2+^ ion (magenta sphere) bridges the crossover phosphates. (**C**) Crossover close-up (representative frame): the Mg^2+^ ion (magenta sphere) coordinated by the crossover A- and C-strand phosphates (thick sticks) and first-shell waters (small red spheres); DNA backbones in pale strand colors, bases in transparent grey. (**D**) Schematic of the sheared base-pair registry (arms named by their constituent strands; arms of 6, 4, 6, and 4 bp; crossover at A/C residues 6|7 and B/D residues 4|5, whose two points are axially displaced by the ≈2.3-Å registry offset reported by Thorpe et al. [22]) and the inter-arm coordinate θ_23_ (arc). (**E**,**F**) The two coaxial stacking isomers: A/C-continuous (dominant in solution, 66–71%) and B/D-continuous (the crystal isomer); exchange between them occurs on the nanosecond scale in the simulations. Panels (**A**,**B**) show the same junction in the crystal and after 200 ns in solution, respectively, superposed onto a common reference frame.

Here we provide that baseline. We simulate the experimental junction with Protein Data Bank (PDB) code 1M6G [22], using parmbsc1, OL21, and OL24 in matched electrolyte of optimal point-charge (OPC) water (16 × 200 ns each)—four strands of d(TCGGTACCGA) forming a stacked-X with a sheared (6/4/6/4) base-pair registry (Figure 1). We quantify the inter-arm-angle landscape against the crystal structure, stacking-isomer populations and exchange kinetics, exchange-point stereochemistry, backbone and sugar-pucker substates, ion occupancy, and the sampling adequacy of the occupancy observables on which the headline comparisons rest.

## 2. Results

### 2.1. System, Stability, and Registry Integrity

**Figure 2:**
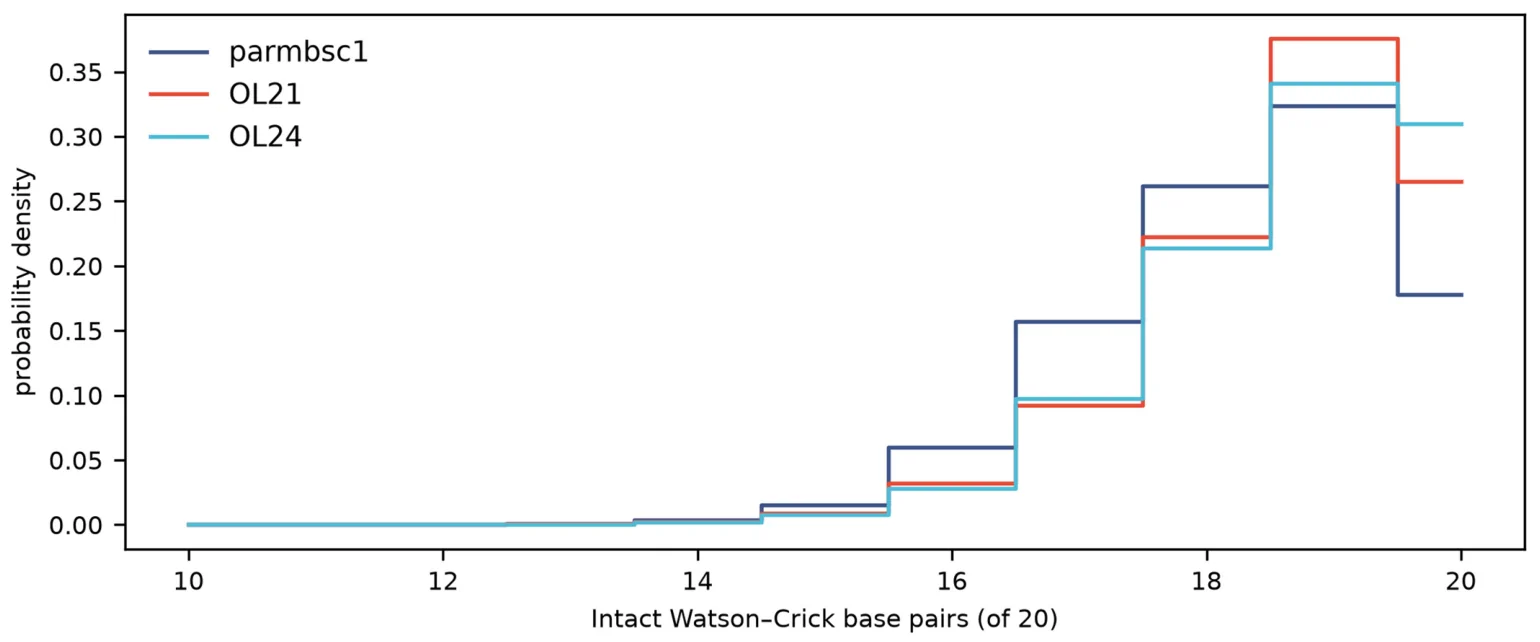
Registry integrity across the 9.6-μs aggregate sampling (48 trajectories; 16 replicas per force field). Distributions of the per-frame number of intact Watson–Crick base pairs (of the 20 pairs of the sheared 6/4/6/4 registry; 3.5-Å heavy-atom criterion): median 19/20 in all force fields; ≥18 pairs in 76–86% of frames; never below 9/20 (the left edge of the plotted range). OL24 holds the fully intact registry (20/20) most often (31.0/26.6/17.8% of frames for OL24/OL21/parmbsc1).

All 48 trajectories (16 per force field) completed the full 200 ns without any integration instability. The junction remained intact throughout: per-frame counts of the 20 registered Watson–Crick base pairs (sheared registry: arms of 6, 4, 6, and 4 bp; Section 4.1) have a median of 19 out of 20 in every force field, stay at ≥18 pairs in 76–86% of frames, and never fall below 9 out of 20 in any single frame of the entire 9.6 μs (each dip below 9/20 is an isolated event from which the registry recovers immediately)—no persistent de-stacking or duplex rearrangement occurs at the 9.6-μs scale. Resolving the registry pair-by-pair shows that no single pair is systematically weak. At the category-mean level, the crossover is the best-protected region: the six junction-proximal pairs (those flanking the two crossover points) average 94–95% intact in OL21/OL24 versus 88–91% for terminal pairs, and transient openings are dominated by terminal-proximal pairs (thousands of sub-ns flickers per pair). Individual pairs dip below their category means—the least-intact pair of each force field retains 63–74% (a junction-proximal CD-arm pair in parmbsc1; a terminal DA-arm pair in OL21 and OL24)—but the crossover remains the most ordered region of the junction, not the least. OL24 holds the fully intact registry (20/20 pairs) most often (31.0% of frames vs. 26.6% for OL21 and 17.8% for parmbsc1; Figure 2). Per-nucleotide dynamics are likewise bounded and force-field consistent (Figure S1): C1′ root-mean-square fluctuations, dominated by the collective opening mode, are smallest at the junction-proximal residues (per-force-field minima ≈ 14–18 Å) and largest at the arm termini (≈22–28 Å); the DNA radius of gyration (2.3–2.6 nm) and the best-fit RMSD to the crystal reference (per-replica medians ≈ 10–28 Å) reach plateaus without drift in all 48 trajectories.

### 2.2. The Inter-Arm-Angle Landscape Is Flat and Open-Dominated in All Force Fields

**Figure 3:**
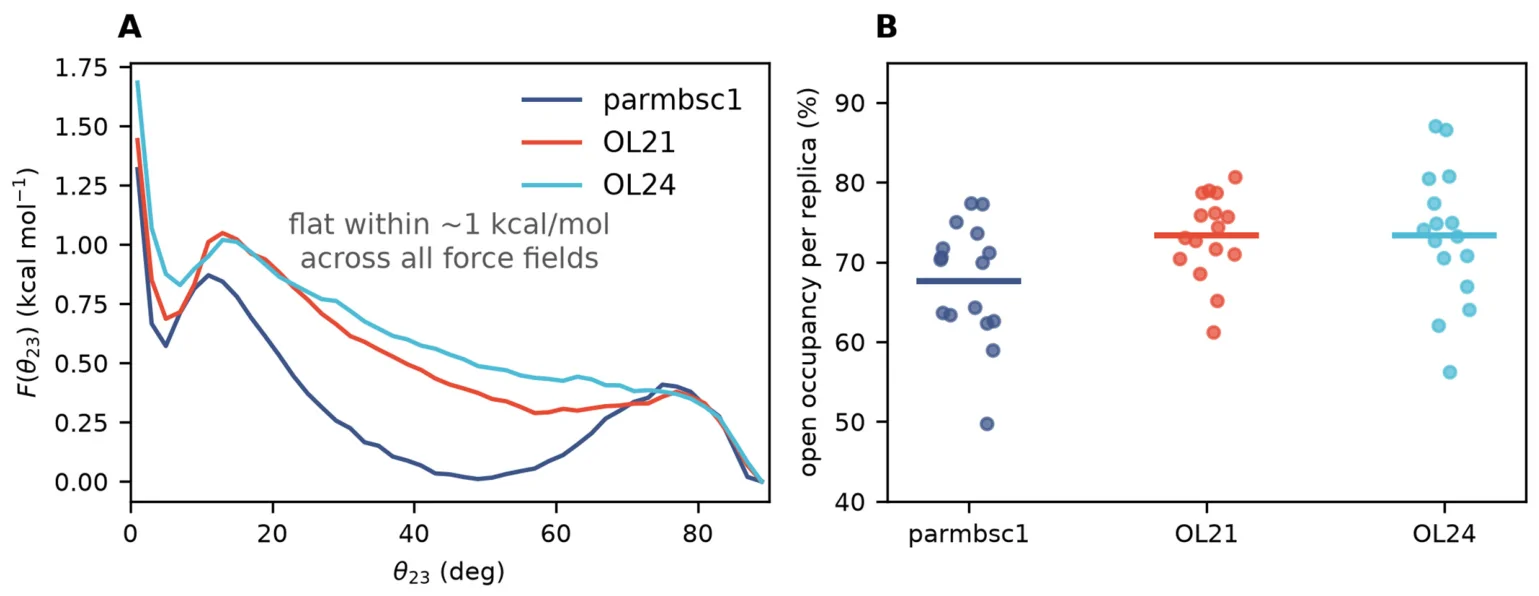
The θ_23_ landscape in all force fields. (**A**) Pooled θ_23_ PMFs (16 × 200 ns per force field; 2° bins; each profile offset by its minimum): the F < 1 kcal/mol region spans θ_23_ = 2–90°, and the pooled-distribution mode is ≈90° in every case. (**B**) Open-state occupancy (θ_23_ ≥ 40°) per replica; bars show per-force-field means (16 replicas). parmbsc1 is 5.7 percentage points less open than OL21/OL24; OL21 and OL24 are indistinguishable (Welch’s *t*-test, *p* = 0.99).

The θ_23_ opening coordinate—the acute angle between the two junction-flanking arm axes (Section 4.4; the complementary crystal-anchored estimator, the IDA, is introduced in Section 2.3)—spans the full 2–90° range within F < 1 kcal/mol in all three force fields, with the most probable value at ≈90°—the junction behaves as a soft X whose opening angle is only weakly encoded by the force field (Figure 3A). Replica-bootstrap 95% confidence bands (1000 resamples; Figure S2) put the uncertainty at 0.39–0.56 kcal/mol at the mode, and split-half re-analysis (first vs. second 100 ns of every trajectory) changes free-energy values by at most 0.84 kcal/mol for all θ_23_ with F(θ_23_) > −1.5 kcal/mol. On a landscape this flat, the mode position is itself an ill-defined statistic (split-half modes can jump by ≈38° within <0.6 kcal/mol), so all conclusions below are phrased in terms of distribution widths and state weights rather than mode locations. The force-field comparison can be compressed into a single per-pair bound: the pairwise free-energy difference ΔF(θ_23_) between two force fields never exceeds 0.42 [0.24, 0.61] kcal/mol (parmbsc1 vs. OL21), 0.55 [0.33, 0.78] (parmbsc1 vs. OL24), and 0.31 [0.15, 0.64] (OL21 vs. OL24) anywhere in the populated range (95% bootstrap bounds of the maximum over θ_23_ of each force-field pair’s ΔF). Correspondingly, the ensembles overlap almost completely across all five compared features: histogram overlap coefficients are 0.83–0.97 for IDA, 0.78–0.94 for the packing distance, 0.85–0.95 for θ_23_, 0.94–1.00 for the isomer variable, and 0.68–0.90 only for χ—the one coordinate with a visible force-field component. The open-dominated, force-field-insensitive picture thus does not hinge on the θ_23_ estimator: the IDA readout (Section 2.3) shows the same broad, mutually overlapping ensembles. The open substate (θ_23_ ≥ 40°) is dominant in all cases, with per-replica occupancies (mean ± s.d.) of 67.6 ± 7.4% (parmbsc1), 73.3 ± 5.3% (OL21), and 73.3 ± 8.4% (OL24) (Figure 3B). The threshold itself is crossed 7–9 times per ns in every force field: the coordinate is a soft, rapidly fluctuating reaction coordinate, not a two-state switch (cf. the isomer exchange below). In free-energy terms, the open state is favored in every force field, only more so for the OL-series (Table 1)—the thermodynamic hierarchy matches the geometric one: a small parmbsc1-vs-OL shift of ≈0.2 kcal/mol and no OL21/OL24 difference. For comparison, an assembly-defective junction variant (a mis-assembled topology generated during an early phase of this work; archived trajectories deposited with this study; Figure S3) showed a qualitative force-field-driven relocation of the θ_23_ PMF minimum (modes 21°/23°/67° for parmbsc1/OL21/OL24); a re-analysis of those archived trajectories reproduces exactly those modes, and on the experimental junction this relocation is not observed.

ΔΔG(parmbsc1 − OL21) = +0.16 kcal/mol (Welch’s *t*-test, *p* = 0.017); ΔΔG(parmbsc1 − OL24) = +0.18 (*p* = 0.043); ΔΔG(OL21 − OL24) = +0.02 (*p* = 0.83). The two ΔG estimators (replica-mean vs. pooled-frame) agree to ≤0.03 kcal/mol. Occupancy contrasts: Hedges’ g = 0.88 (parmbsc1 vs. OL21) and 0.72 (parmbsc1 vs. OL24); the parmbsc1 contrasts retain significance when the slowest parmbsc1 replica (replica 5) is excluded (−4.5 pp, *p* = 0.034) and strengthen when the two slowest OL24 replicas are excluded (−7.5 pp, *p* = 0.008).

### 2.3. The Crystallographic Stacked-X Geometry Is a Minor State in Solution

**Figure 4:**
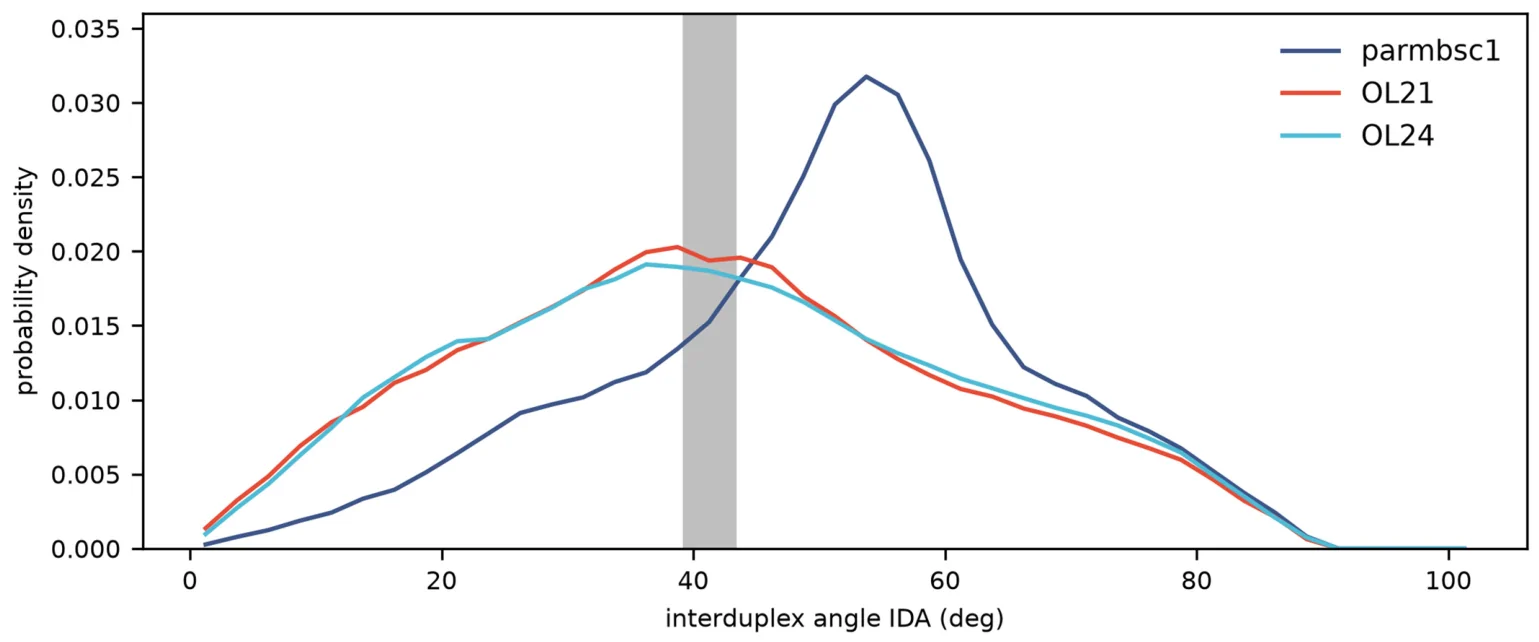
Interduplex-angle distributions vs. the crystal anchor. Simulated IDA medians are 51.9° (parmbsc1), 40.9° (OL21), and 41.3° (OL24); the shaded band marks the 1M6G crystallographic crossover angles (39.3° Ca^2+^/Na^+^ form, 43.3° Sr^2+^ form). Crystal-like frames (IDA within this interval) account for 2.2–4.6% of simulation frames.

Compared against the crystal anchor, the IDA (Section 4.4) of the simulated ensembles has medians of 51.9° (parmbsc1), 40.9° (OL21), and 41.3° (OL24), with interquartile ranges of 40–60° (parmbsc1) and 28–56° (OL21/OL24) (Figure 4). The crystallographic crossover (interduplex) angles of 1M6G are 39.3° in the Ca^2+^/Na^+^ form and 43.3° in the Sr^2+^ form [22]; simulated IDA values fall within the interval they span in only 2.2–4.6% of frames. Our estimator returns 45.4° on the crystal coordinates themselves (a ≈2° method difference), so this fixed window is conservative with respect to the estimator offset. The comparison extends beyond the angle to the crystal’s ion sites: mapping the five crystallographic Sr^2+^ positions onto every simulated frame (per-frame best-fit alignment), the sites are water-occupied 93–97% of the time (water criterion) and host a Na^+^ within 4.5 Å in only 6–10% of frames (independent ion criterion; Figure S4)—above the bulk random expectation of ≈4.6% (a 4.5-Å sphere at the bulk Na^+^ density), but far from full occupancy. The divalent-cation spine that organizes the crystal lattice is thus a solution-invisible site: in solution, the strong cation site is the crossover phosphates themselves (see below), not the minor-groove spine.

Two caveats frame this comparison: the crystal value reflects a lattice-stabilized, divalent-cation-organized state, and the isolated-junction solution ensemble probed here (≈0.20 M NaCl, 5.9 mM MgCl_2_ after equilibration) is not under lattice pressure. Our conditions (≈0.20 M NaCl plus 5.9 mM MgCl_2_) lie in the ionic regime where FRET measurements on longer constructs report the stacked-X as the dominant species [18,19,20]; our minimal junction is open-dominated under the same class of conditions, and the open-dominated ensemble must therefore be interpreted with care against this FRET solution literature. We flag this tension explicitly rather than resolve it. The candidate explanations are construct design (a 10-mer with 4–6 bp arms has a small stacking surface, and its termini adjoin the crossover), the sheared registry, and possible force-field over-opening; we state the first as our working hypothesis without claiming generality.

As a concrete bridge to these experiments, we computed the site-separation distributions between the four arm-terminal reference positions (C1′ atoms; all 9.6 μs; Figure S5A) and converted them to predicted FRET efficiencies with a Cy3/Cy5-typical Förster radius (R_0_ = 5.4 nm) [39], assuming isotropic dye orientation, E = 1/(1 + (R/R_0_)^6^). The six arm–pair separations have medians of only 2.6–3.8 nm—squarely below R_0_, on the nearly flat short-distance shoulder of the FRET efficiency curve E(R)—so the predicted efficiencies are uniformly high (per-pair medians 0.89–0.99 for every force field; Figure S5B): terminal-dye FRET on this minimal construct is conformation-insensitive and cannot resolve the open ensemble. Discriminating stacked from open states by FRET requires dye separations on the steep, distance-sensitive flank of E(R): the classic single-molecule FRET constructs achieve this through longer arms and dye positions chosen for distance sensitivity (e.g., McKinney et al. [3]), whereas the terminal sites of our 4–6 bp arms cannot—reinforcing the construct-design working hypothesis above. Within the simulated ensemble, the crystal-like geometry (interduplex angle in 39.3–43.3°) is real but minor (2–5% of frames), and opening does not disrupt the stack. In the standard Watson–Hays–Ho geometry language [24], the additional interduplex parameters are equally soft: the J-roll junction roll (four-center dihedral across the crossover) has circular medians of 138–168° with circular s.d. of 63–72°, and the J-slide axial offset has medians of 4.9–7.0 Å (not to be confused with the fixed ≈2.3-Å registry offset of the crossover points; Section 4.1)—both far from any single crystallographic value, confirming that the solution ensemble explores a broad family of crossover geometries of which the crystal fixes one member. Table 2 assembles the principal structural parameters against their crystallographic anchors.

### 2.4. Opening Is Not De-Stacking

A central mechanistic question is whether the broadly open ensembles are stacked or de-stacked. The strict test is the axial stacking step: the projection of the junction-adjacent base-pair-center separation onto the stack axis of the frame’s own isomer registration, whose canonical B-DNA value is ≈3.4 Å. This step has medians of **3**.2 Å (parmbsc1), 3.7 Å (OL21) and 3.4 Å (OL24) (Figure S4), and within each force field the open and compact substates differ by at most 0.5 Å (open/compact: 3.2/3.2, 3.6/4.1, 3.4/3.4 Å for parmbsc1/OL21/OL24)—by the true coaxial measure, the junction remains stacked to within a canonical B-DNA rise whether it is open or compact. The junction-proximate base pairs are also tightly packed around the exchange point in every force field (pair-center distances 3.5–4.2 Å, unchanged between open and compact frames; 78.9–80.9% of open frames within the 6.5-Å packing criterion vs. 74.7–83.9% of compact frames; the joint θ_23_ × packing-distance PMF is shown in Figure S6). The junction therefore opens like a pair of pliers around an intact, coaxially stacked core: opening and de-stacking are distinct processes, and the solution-vs-crystal difference identified above is a difference in inter-arm angle, not in stacking. The two crossover elbows (the two sides of the sheared registration, quantified by the same junction-adjacent pair-center distances) are only weakly asymmetric (median 3.5 vs. 4.2 Å in parmbsc1; ≤0.1 Å in OL21/OL24), and the isomer assignment separates the registrations: frames assigned A/C-continuous keep their elbow centers at 3.8 Å on average, B/D-continuous frames at 5.5–6.3 Å—a geometric difference between the two crossover registrations (the pair-center distance conflates stacking rise with helical twist), consistent with the different phosphate exposure of each isomer. Normalized mutual-information analysis across θ_23_, χ, packing distance, isomer assignment, and Mg^2+^-bound status confirms that the coordinate set is nearly uncoupled: no pairwise coupling exceeds 0.10 (in I/min(H_1_,H_2_) units), with the strongest being packing–Mg^2+^ and χ–Mg^2+^—the ion senses the local geometry weakly but does not drive it.

### 2.5. Stacking-Isomer Exchange Is Force-Field Independent

**Figure 5:**
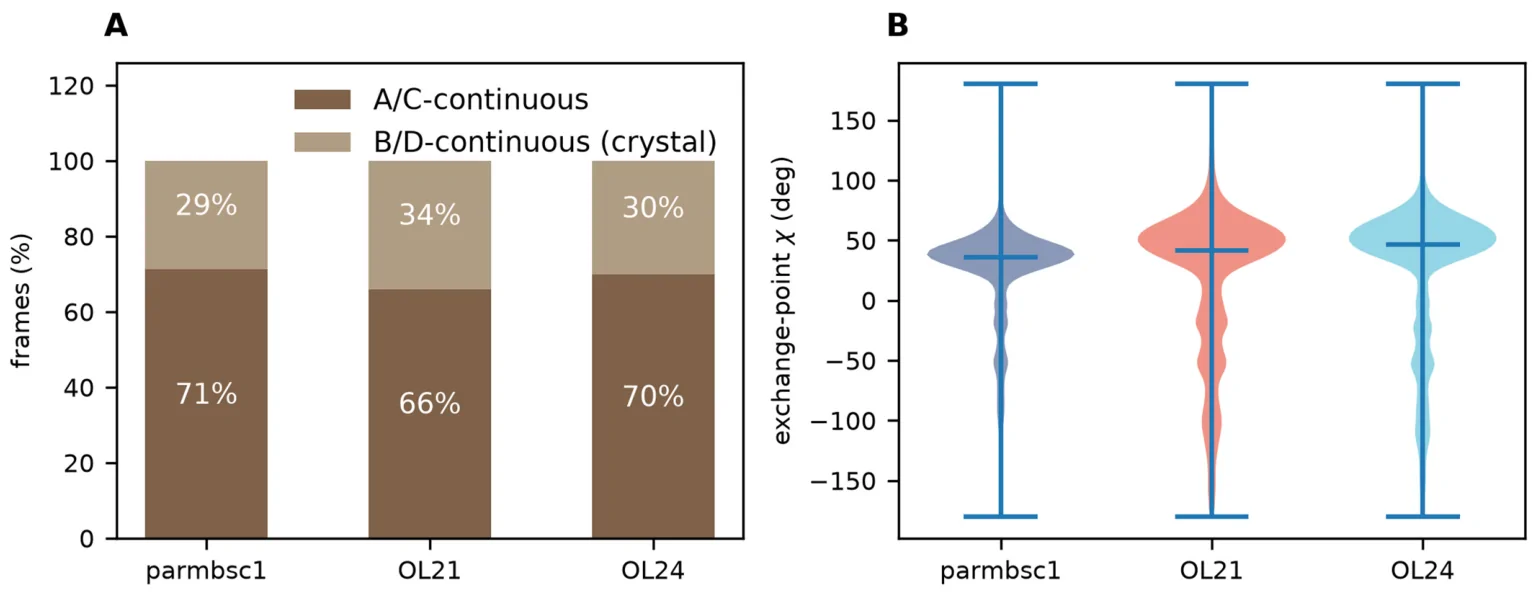
Stacking isomers and exchange-point stereochemistry. (**A**) The A/C-continuous isomer dominates in all force fields (66–71%) over the B/D-continuous crystal isomer. (**B**) Exchange-point pseudodihedral χ: linear medians advance monotonically 35.8° → 41.6° → 46.3° (parmbsc1 → OL21 → OL24; circular s.d. 44–61°); circular medians 30.1°/34.2°/40.7°.

When each frame is assigned to a stacking isomer by the minimal-angle criterion of Section 4.4, the A/C-continuous isomer dominates in all force fields (71.2%, 66.0%, 69.8% for parmbsc1, OL21, OL24; bootstrap 95% confidence intervals (CIs) on the A/C fraction: 66–76%, 62–70%, 66–74%) over the B/D-continuous isomer—the one observed in the 1M6G crystal (Figure 5A). Both isomers are substantially populated, confirming stacked-isomer exchange in every force field, with force-field differences of ≤5.2 pp—far smaller than the isomer gap itself. The exchange kinetics are likewise force-field independent: after temporal smoothing of the assignment (0.5 ns majority filter; the raw minimal-angle assignment flickers frame-to-frame when the two candidate stackings are near-degenerate), the A/C ↔ B/D exchange rate is 0.50 ± 0.12, 0.57 ± 0.14, and 0.53 ± 0.11 ns^−1^ in parmbsc1, OL21, and OL24 at the 0.5 ns filter (mean ± s.d. across the 16 replicas). The rate depends on the filter width (0.90–0.98 ns^−1^ at 0.25 ns; 0.30–0.35 ns^−1^ at 1.0 ns); it is therefore partly convention-defined—residual misassignments can only add spurious transitions—and should be read as an upper bound on assignment-defined exchange. The force-field independence holds at every filter width. This is six orders of magnitude faster than the millisecond isomer exchange measured by single-molecule FRET on Mg^2+^-folded junctions [3,5]—an expected consequence of the much smaller stacking surface of the 4–6 bp arms, the high monovalent salt, and the absence of lattice context—and it should be read as a property of this minimal system rather than a force-field prediction for origami-embedded crossovers. Consistently, a three-state decomposition (compact-packed/open-packed/open-splayed; Section 4.4) yields open-packed-dominated weights—compact-packed 27–32%, open-packed 64–70%, open-splayed 3.5–4.1% (95% bootstrap CI 1.8–5.5% over force fields; ≈700–800 frames per replica and 11,000–13,000 pooled frames per force field)—and implied slow relaxations of only 0.04–0.05 ns (four to five frame intervals); there are no metastable substates beyond the broad, flat landscape itself.

### 2.6. Exchange-Point Stereochemistry: A Small, Monotonic OL24 Shift

The junction pseudodihedral χ (explicit atom ordering; Section 4.4) has broad distributions in all force fields (circular s.d. 44–61°), but the circular medians advance monotonically: 30.1° [bootstrap CI 25.8, 33.5] (parmbsc1) → 34.2° [28.1, 39.1] (OL21) → 40.7° [34.3, 46.2] (OL24) (linear medians 35.8°/41.6°/46.3°, quoted for comparison; Figure 5B; full circular distributions in Figure S7)—a displacement of ≈+10° from parmbsc1 to OL24, for which the parmbsc1 and OL24 confidence intervals are just disjoint. Conditioned on the θ_23_-defined open and compact substates, χ shifts by at most 2.7°, establishing it as an independent, force-field-encoded signature of the exchange point rather than a correlate of the opening coordinate (joint θ_23_ × χ PMFs in Figure S8). Given the breadth of the distributions and the convention-dependence of the sign, we report this as a descriptive distributional shift and do not map χ onto the left/right-handed nomenclature.

### 2.7. Backbone Substates: OL24’s Sugar-Pucker Design Target Is Visible at the Junction

Per-residue sugar pucker (Altona–Sundaralingam pseudorotation; Section 4.4) shows South (C2′-endo) dominance in all force fields—and a clear OL24-specific shift: the South fraction is 0.924 (parmbsc1), 0.906 (OL21), and 0.799 (OL24), i.e., OL24 shifts ≈12 pp of the population toward North (C3′-endo, A-form-like) character (Table 3). This is precisely the degree of freedom OL24’s ν_0_–ν_4_ refinement targets [29], observed here on an experimental junction. The BI/BII equilibrium (canonical ε/ζ backbone substates; Section 4.4) differentiates OL21 and OL24 from parmbsc1 (BII fractions 0.35/0.33 for OL21/OL24 vs. 0.23 for parmbsc1, overall) and is enriched at the junction residues in OL21 (0.412) and OL24 (0.378) relative to parmbsc1 (0.226); the crystal reference at the junction is 0.50 (per-residue fractions in Table S3). Junction residues show slightly reduced South character in parmbsc1 and OL21 (0.901 and 0.895 vs. 0.924 and 0.906 overall) and slightly increased South character in OL24 (0.818 vs. 0.799); in all cases, the deviation from the helical mean is small, consistent with local distortion at the crossover.

### 2.8. The Single Mg^2+^ Occupies the Crossover Pocket in All Force Fields

**Figure 6:**
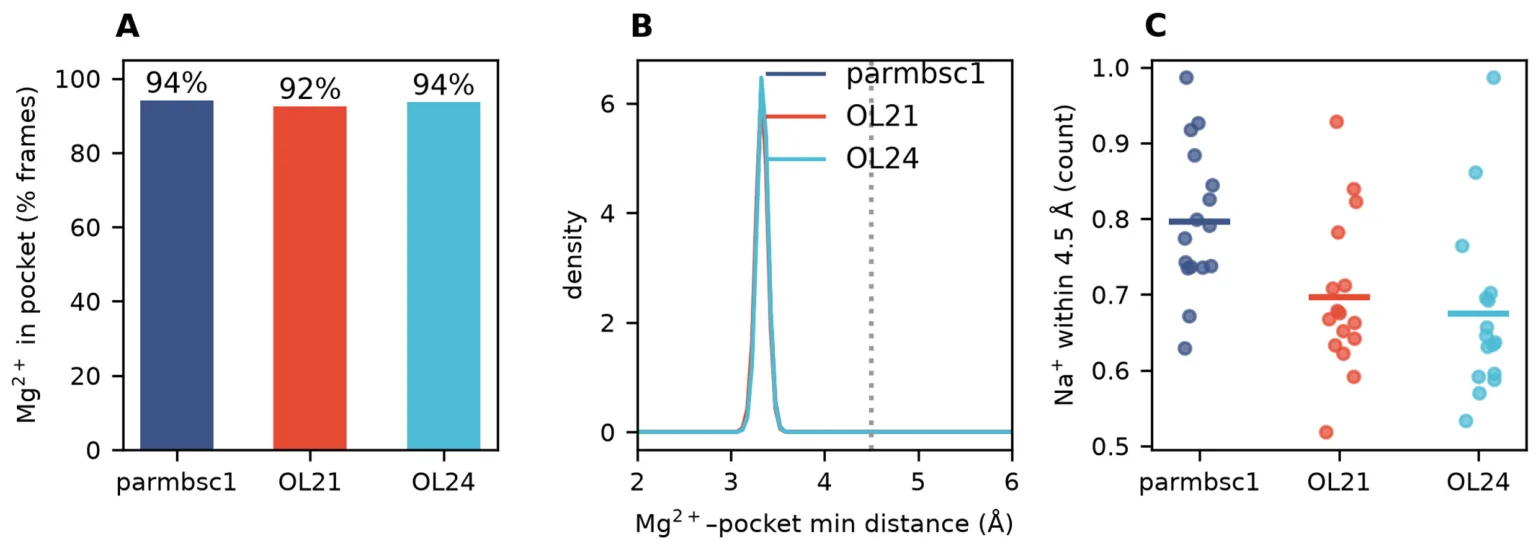
Crossover ion occupancy. (**A**) The single Mg^2+^ (5 mM) resides within 4.5 Å of the eight boundary phosphates in 92–94% of frames in every force field. (**B**) Mg^2+^–pocket minimum-distance distributions (inner-sphere ≈3.3 Å; axis shown to 6 Å—the remaining ≈1% of frames extend the tail to ≈70 Å). (**C**) Na^+^ count within 4.5 Å (Welch’s t-test on per-replica means, n = 16 per force field): parmbsc1 > OL21 (*p* = 0.008), parmbsc1 > OL24 (*p* = 0.003); OL21 ≈ OL24.

At a MgCl_2_ level typical of origami buffers (5 mM nominal; one Mg^2+^ in the simulation cell), the magnesium ion resides within 4.5 Å of the eight boundary phosphates in 92.5–94.1% of frames in every force field (Table S2; per-force-field bootstrap CI within ±5 pp of the mean). The occupancy is insensitive to the contact cutoff between 4.0 and 5.0 Å because the bound ion sits at inner-sphere Mg–P distances of ≈3.3 Å on average (minimum observed 3.0 Å; Figure 6A,B). The residence pattern separates threshold flicker from true dissociation. Of the ≈220–290 boundary exits per trajectory per force field (per-replica range 54–586), only ≈90–130 are sub-threshold flickers (peak separation < 6 Å or < 20 ps); the remaining ≈130–170 events per 200 ns (mean ± s.d. across replicas) are genuine pocket escapes that leave beyond 6 Å for ≥ 20 ps—an escape rate of ≈0.63–0.85 ns^−1^, with median escape duration ≈40 ps and a tail to ≈1.3–1.5 ns (the classification is robust for escape thresholds of 5.5–7 Å). After each escape, the ion returns to the pocket rather than diffusing away: the longest continuous 4.5-Å residence in any trajectory is 156–173 ns (cross-replica maxima per force field; median per-replica maximum 57–71 ns; individual replicas range down to 22 ns), and inner-shell chelation exchanges faster still (≈310–350 exits from the 3.5-Å coordination per 200 ns, median excursion ≈10 ps). The ion is bound but its hydration shell is dynamic: the first 3.5-Å shell holds a median of ≈3.8–3.9 waters (alongside ≈4 phosphate oxygens—an 8-coordinate, fully phosphate-chelated state that is a known fingerprint of the Li–Merz CM 12-6 divalent parameterization [40,41] and should be read as parameterization-conditional), and individual waters exchange in and out at 4.9–6.0 events/ns, with individual residence times up to 72–83 ns (Figure S9). The chelation is strikingly site-specific: in 94–95% of chelated frames the Mg^2+^ bridges exactly the A- and C-strand phosphates—the two strands that are continuous in the dominant A/C isomer—with minor A-only or C-only states making up the remainder. Na^+^ is a secondary pocket occupant: 0.67–0.80 ions within 4.5 Å on average, with parmbsc1 binding significantly more than both OL21 (*p* = 0.008) and OL24 (*p* = 0.003), while OL21 and OL24 are indistinguishable (Figure 6C; junction-frame water and Na^+^ number-density maps in Figure S10). Within each force field, the mean Na^+^ count within 4.5 Å of the pocket is essentially independent of the open/compact substate (Δ ≤ 0.05 ions), i.e., the ions follow the junction rather than driving its opening. This Mg^2+^-over-Na^+^ hierarchy mirrors the cation competition measured for duplex DNA [42]. This robust, site-specific Mg^2+^ crossover binding is consistent with the experimental role of Mg^2+^ in stabilizing folded junctions [43]. Notably, it is a different site from the crystallographic divalent-cation spine (above), and it inverts the behavior obtained in the earlier assembly-defective system, where the Mg^2+^ never visited the crossover. On an experimental junction, the crossover is the first site the ion finds.

### 2.9. Sampling Adequacy: The Pooled Ensembles Are Statistically Robust

**Figure 7:**
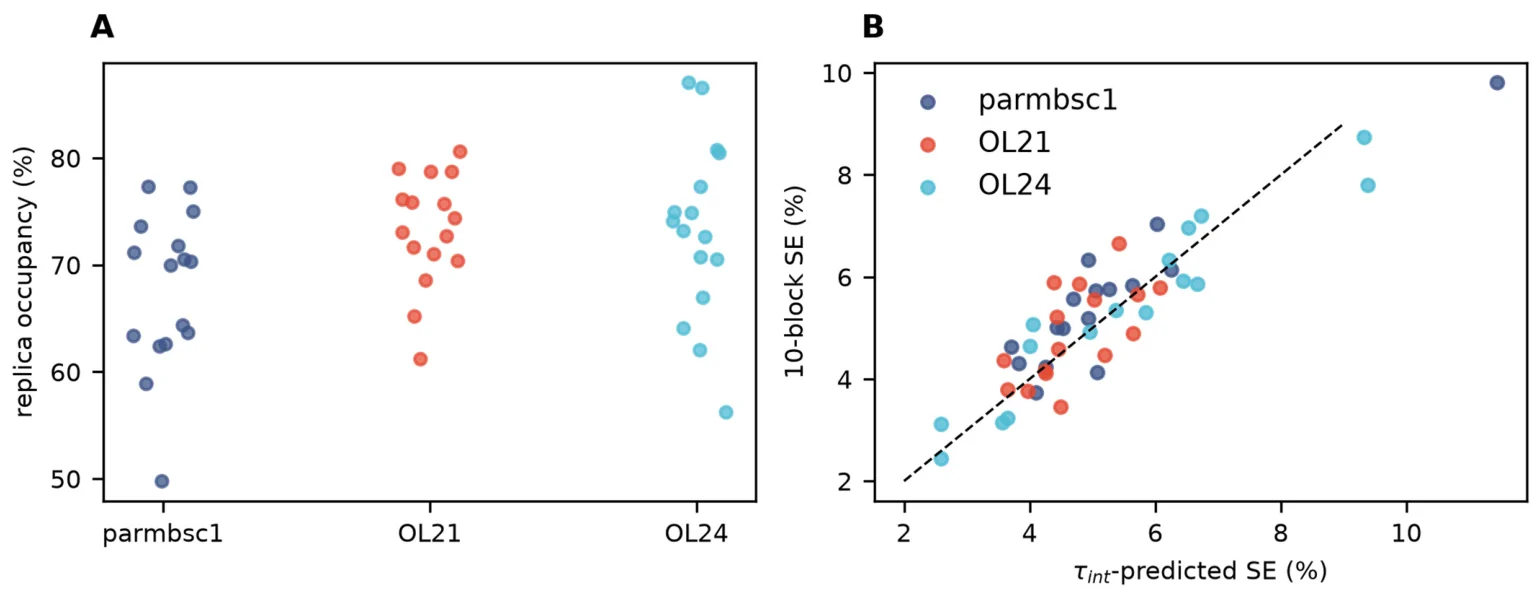
Sampling adequacy. (**A**) Per-replica open occupancy: replica spread is consistent with within-trajectory noise (between-replica effective correlation scale 1–4 ns; no heterogeneity term). (**B**) 10-block occupancy standard errors vs. the Sokal τ_int_ prediction √(2τf(1−f)/T) for all 48 replicas; per-force-field mean ratios 1.00–1.07 (per-replica span 0.77–1.34); dashed line: identity.

All autocorrelation estimates use the Sokal automatic-window procedure (c = 5; Section 4.5) [44,45] on the binary open-state occupancy coordinate. Median integrated autocorrelation times τint are short—1.11 ns (parmbsc1), 1.09 ns (OL21), 1.66 ns (OL24); max 5.2 ns—giving a median of 61–92 statistically independent occupancy samples per 200 ns replica (per-replica span 19–173; per-replica statistics in Table S1). The sampling checks specified in Section 4.5 pass cleanly here. The 10-block occupancy standard errors match the τint prediction √(2τf(1−f)/T)—per-force-field mean ratios of 1.00 (OL24), 1.04 (OL21) and 1.07 (parmbsc1), with per-replica values spanning 0.77–1.34 (Figure 7B). The disjoint-window drift (the absolute difference between the mean occupancies of the 20–110 ns and 110–200 ns windows) has per-force-field medians of 11.1, 13.0 and 8.5 pp (single-replica maximum 35.4 pp). Critically, the between-replica variance corresponds to an effective correlation scale of only 1–4 ns, that is, within-trajectory noise with no long-lived replica basins at the 200 ns scale—a replicability check long advocated for DNA simulations [46]. The 16-replica design nevertheless has limited power to exclude a mild heterogeneity component (Figure 7A). The pooled force-field occupancies therefore carry ≈±1.3–2.1 pp standard errors on the mean, two- to fourfold below the largest reported force-field contrast, and the force-field insensitivity of the θ_23_ landscape is a positive, protocol-validated finding rather than an artifact of insufficient sampling. For completeness, we state the design’s statistical power: with 16 replicas per force field, the minimal detectable occupancy contrast (80% power, α = 0.05) is 7.4 pp (pooled s.d. 7.5 pp), and the minimal detectable ΔG is 0.22 kcal/mol—the parmbsc1-vs-OL-series contrasts we report (≈5.7 pp, ≈0.2 kcal/mol) are at the edge of this design’s resolution and are explicitly not equated with zero. As practical reuse criteria, the values this study met were: per-replica block-SE/τint ratios within 0.77–1.34 of unity (per-force-field means 1.00–1.07), disjoint-window drift medians of 8.5–13 pp (single-replica maximum 35.4 pp), and a between-replica effective correlation scale no larger than a few nanoseconds; ensembles that violate these bounds by wide margins warrant longer trajectories or more replicas before force-field contrasts are interpreted.

## 3. Discussion

On an experimental Holliday junction, OL24 left the junction’s principal conformational observable—the inter-arm-angle ensemble—essentially unchanged relative to OL21 (0.0 percentage-point occupancy difference) and altered the parmbsc1 ensemble by only ≈6 percentage points. What OL24 did change were local, chemically specific degrees of freedom: the sugar-pucker equilibrium, precisely the torsion it was designed to correct [29], and the exchange-point pseudodihedral (+10° vs. parmbsc1, +5° vs. OL21). For origami-scale simulations, in which crossovers are embedded in a strained lattice, such local pucker and stereochemistry shifts could still accumulate. Mechanistically, a pucker shift at the crossover-adjacent nucleotides alters the local helical twist and rise, and under the torsional and tensile constraints of a lattice, these small per-crossover offsets cannot relax freely; across the hundreds of crossovers of a nanostructure they could bias the global twist and the inter-crossover distances. A direct, verifiable test is to simulate the same junction design embedded in a constrained origami tile under a designed global twist with OL21 versus OL24: accumulation would appear as a force-field-dependent drift in per-crossover pitch and inter-crossover distances, a regime reachable with coarse-grained models parameterized from baselines such as the present one [47]. However, the present results remove the concern that junction-level force-field choice qualitatively reorganizes the crossover ensemble of an isolated junction.

Zhang et al. [31] reported that OL15 over-stabilizes the closed/stacked junction by 5–6 kcal/mol in the longer-armed J1 system. Our 10-mer junction showed no such closed dominance—its ensemble was flat and open-dominated—and the crystallographic stacked geometry was a minor state. This contrast likely reflects system design (arm length, sequence, terminal context) rather than a contradiction: stacking surface and terminal-fraying economics differ sharply between 20-mer arms and 4–6 bp arms. The concurrent topology-PMF study by Lemmens et al. [32] reached a complementary conclusion at the topology level (antiparallel global minimum; parallel < 1%), consistent with our finding that the junction remained intact and antiparallel throughout. Singh et al. [33], simulating the same sequence, reported site-selective ion binding affecting conformation; our ion analysis localizes that picture: one Mg^2+^ permanently bridges the crossover phosphates in all force fields, with Na^+^ secondary. The present study adds the missing parmbsc1 baseline, the crystallographically anchored IDA coordinate, the local backbone readout that reveals OL24’s design signature, and an explicit sampling-error validation.

Two of our results speak directly to how proteins read Holliday junctions. First, the junction is a stacked, coaxially paired entity across its entire conformational landscape—even wide-open frames retain canonical stacking steps—which argues that the stacked architecture, not a planar form, is what repair machinery encounters in a high-salt, Mg^2+^-containing environment; open-planar recognition such as that reported for BRCA1 [6] would then require active deformation of a stacked junction, or protein conditions that shift the ensemble. Second, the Mg^2+^ ion bridging the A/C-strand phosphates marks one of the two possible registrations with a bound divalent cation; since the A/C-continuous isomer dominates in solution while the B/D registration is the one crystallized, metal-dependent stabilization of one registration is a plausible (and testable) contributor to isomer selection by resolvases and other junction-binding proteins [2,7]. Both statements are force-field independent at the resolution of this study.

For DNA nanotechnology, the practical implications are threefold. First, for isolated-junction properties, force-field choice among parmbsc1, OL21, and OL24 is a second-order uncertainty compared with ion environment and system context; benchmarking effort is better spent on ion parameterization (our Mg^2+^ result is parameterization-conditional) and on junction-in-lattice validation (e.g., against cryo-EM structures of origami objects [48] and atomistic in situ benchmarks [9]). Second, the crystallographic stacked-X geometry should not be used as the reference state for solution simulations of short-junction motifs: the solution ensemble measured here is broadly open, and the crystal state is reproduced as a minor population (2–5%) rather than the dominant one. Third, the conformational baseline established here—broadly open, coaxially stacked, Mg^2+^-bridged—provides the structural starting point for simulation-guided design of crossover-containing nanodevices [47], from wireframe origami scaffolds for drug encapsulation to HJ-based biosensor elements, and the validated sampling protocol lowers the barrier for such simulations by providing a quantitative criterion for when the ensemble is adequately sampled. The Mg^2+^ crossover bridge characterized here also provides a candidate molecular-level rationale for an empirical cornerstone of origami practice: DNA nanocontainers and origami devices are folded and stabilized in Mg^2+^-containing buffers, and the site-specific, force-field-independent crossover binding we observed (92–94% occupancy at 5 mM MgCl_2_; Section 2.8) is the single-crossover counterpart of the ion-mediated stabilization those conditions require. For origami crossovers—where topology constrains the junction into the stacked-X family—our results predict that the crossover ensemble will be dominated by the compact-stacked branch of the landscape, but the ≈2:1 isomer preference measured here (A/C over B/D in solution, inverted with respect to the crystal) suggests isomer populations may be environmentally controlled and should be re-examined in origami context.

Several limitations frame the scope of these conclusions: a single symmetric sequence (10-mer, sheared registry inherited from the crystal); isolated junction rather than an origami lattice; no terminal restraints; DNA hydrogens unconstrained at dt = 2 fs; fixed protonation; the χ sign convention is not calibrated to literature handedness nomenclature; ion results are conditional on the OPC-matched Li–Merz sets (HFE Na^+^, CM Mg^2+^) [40,49]; and the crystallographic IDA comparison is indirect (lattice context differs).

## 4. Materials and Methods

### 4.1. System

Four strands of d(TCGGTACCGA) (10 nt each), taken from the 1.65-Å crystal structure 1M6G [22] (biological assembly; four copies of the decamer; Sr^2+^ and waters removed, hydrogens added with tleap (AmberTools 26 [50]), were converted to GROMACS format with a ParmEd-based converter script (ParmEd 4.3.1). The junction carries the sheared base-pair registry of the crystal (arms of 6, 4, 6, 4 bp; crossover at A/C residues 6|7 and B/D residues 4|5). The DNA strands were solvated in OPC water [51] with 68 Na^+^, 34 Cl^−^ and 1 Mg^2+^ (net charge zero; nominal 0.15 M excess NaCl and 5 mM MgCl_2_). Final systems: 36,607 atoms for each force field; the three topologies differed only in the DNA parameter set. The single Mg^2+^ was placed in bulk solution at system construction.

### 4.2. Force Fields, Water, and Ions

parmbsc1 [26], OL21 [28], and OL24 [29] were used with the OPC water model and the OPC-matched Li–Merz 12-6 ion set (Na^+^/Cl^−^ from the hydration-free-energy (HFE) monovalent parameterization [49]; Mg^2+^ from the CM divalent parameterization [40]). The OL24 validation used TIP3P/OPC water and prescribes no single water model [29]; we kept the water model and ion parameters identical across force fields so that the DNA parameter set was the only systematic difference in this study, at the documented cost that the parmbsc1 baseline ran outside its original validation pairing (the OL21 versus OL24 comparison, which is the focus of this study, is unaffected). Mg^2+^ interactions were thus carried by the OPC-matched CM 12-6 parameters under PME long-range electrostatics (Section 4.3); the observable consequences of this parameterization for ion binding are quantified in Section 2.8.

### 4.3. Simulation Protocol

Each system was energy-minimized and equilibrated by annealed NVT (0.5-fs timestep for 20 ps, 1 fs for 25 ps, 2 fs for 50 ps) followed by two-stage NPT equilibration (Berendsen [52], then Parrinello–Rahman [53]). Production runs of 200 ns were performed with 16 replicas per force field using a 2-fs timestep, PME [54] on a 1.0 nm grid, Verlet cutoffs with an energy–pressure dispersion correction, a stochastic velocity-rescaling thermostat [55] (τ = 0.1 ps, 300 K), and a Parrinello–Rahman barostat (τ = 2.0 ps, 1 bar); coordinates were saved every 10 ps. Water was kept rigid with SETTLE [56], whereas DNA and ions were fully flexible with no bond constraints—a documented deviation from the common hydrogen-bond-constrained setup, applied identically across all force fields. Ensemble fidelity was supported by total-energy drifts of ≤0.001 kJ mol^−1^ ns^−1^ and mean temperatures of 300.0 K across all 12 sampled production segments (three force fields), as well as zero constraint incidents over 48 × 200 ns. Replicas shared initial coordinates and velocities from the equilibrated state and differed only in the v-rescale random seed (unique per replica); pairwise DNA C1′ differences exceeded 6 Å within 1 ns. All simulations were performed using GROMACS 2026.3 [57].

### 4.4. Coordinates and Observables

Arm axes are the first principal components of arm C1′ atoms (arms of 12, 8, 12, 8 C1′ atoms per the sheared registry; junction-proximal subsets were also checked for robustness). θ_23_—a coordinate defined herein for this junction’s registry (Figure 1D; the subscript is a fixed label, not an arm index)—is the acute angle between the principal axes of the two junction-flanking 4 bp arms, BC and DA; the open/compact partition used below is the operational threshold θ_23_ ≥ 40°. The IDA operationalizes the literature interduplex-angle concept [24] as follows: for each of the two candidate stackings (A/C-continuous vs. B/D-continuous; the crystal adopts B/D), each stack is the arm pair joined through the junction, represented by the vector connecting the C1′ centroids of its two arms; the frame’s IDA is the smaller of the two acute angles between the stack vectors, and the stacking that yields it is the frame’s isomer assignment. On the 1M6G coordinates, this estimator returns 45.4° vs. 43.3° reported crystallographically (≈2° method difference). Stacking isomers are assigned by which strand pairs are continuous (A/C vs. B/D); the assignment is smoothed with a 0.5 ns majority filter for kinetic analysis (the raw assignment flickers near degeneracy). The packing criterion counts a frame as packed when both junction-adjacent base-pair-center distances are ≤6.5 Å. The three-state decomposition labels frames as compact (θ_23_ < 40°, packing not resolved), open-packed (θ_23_ ≥ 40° with at least one junction-adjacent pair-center distance ≤6.5 Å), or open-splayed (θ_23_ ≥ 40° with both distances >6.5 Å); histogram overlap coefficients are ∫min(p_1_,p_2_)dx over per-feature histograms (2° θ_23_ bins, 5° χ, 2.5° IDA, 0.25 Å packing bins), and normalized mutual information uses 8×8 quantile bins with I/min(H_1_,H_2_). The exchange-point pseudodihedral χ = dihedral(C1′(A6), C1′(A7), C1′(B4), C1′(B5)) (χ here denotes the exchange-point pseudodihedral, not the glycosidic torsion conventionally denoted χ); sign conventions are definition-dependent and are not mapped to handedness nomenclature. Watson–Crick registry integrity uses a 3.5-Å heavy-atom minimum-distance criterion per registered pair—a pair-explicit inter-strand hydrogen-bond analysis (the role gmx hbond plays for generic systems) in which registry identity, not geometry alone, is scored. The geometric stringency of this criterion was cross-checked against explicit hydrogen-bond geometry: adding the 30° donor–hydrogen–acceptor angle cutoff of gmx hbond to a donor–acceptor-atom-pair variant of the test changes per-pair occupancies by ≤0.3 percentage points for interior and junction-proximal pairs and ≤5.3 points for fraying terminal pairs over all 48 trajectories, and a literal gmx hbond run (default 0.35 nm and 30° criteria) on a representative replica returns pair-intact fractions of 99.7–100% on spot-checked pairs and mean inter-strand hydrogen-bond counts of 15.05/10.37/12.84/10.42 for the AB/BC/CD/DA arms (Watson–Crick bond counts 15/11/15/11). The ion pocket comprises the 8 boundary phosphates; occupancies count ions within 4.5 Å (Na^+^) and the Mg^2+^ minimum distance. Backbone substates: ε/ζ per residue, with BI/BII classified by the sign of the circular difference ζ−ε; sugar pseudorotation P from ν_0_–ν_4_ via the Altona–Sundaralingam relations [58], South = |P − 162°| ≤ 90°. Free-energy profiles are computed as F = −kBT ln P on normalized histograms (T = 300 K), with each profile offset by its minimum; ΔG(open − compact) = −kBT ln[f/(1−f)] from the open-state occupancy f, with replica-mean and pooled-frame estimators.

### 4.5. Sampling Analysis

The integrated autocorrelation time τint was estimated using the Sokal automatic-window method (c = 5) on the binary occupancy coordinate (FFT autocorrelation). The effective sample size was defined as Neff = T/(2τint). Ten-block occupancy standard errors were benchmarked against √(2τf(1−f)/T). Disjoint-window drift was assessed between 20 and 110 ns and 110 and 200 ns, and between-replica variance was converted to an effective correlation scale. Statistical comparisons used Welch’s t-test on per-replica means (n = 16) with Hedges’ g.

## 5. Conclusions

In summary, on the experimental d(TCGGTACCGA) junction (48 × 200 ns; 9.6 μs), the inter-arm-angle ensemble is flat, open-dominated, and remarkably force-field insensitive; the crystallographic stacked-X is a minor solution state; Mg^2+^ occupies the crossover in all force fields; and OL24’s measurable signatures sit exactly where its parameterization changed—the sugar pucker and the exchange point. We release the validated sampling protocol (Sokal τint, block-SE consistency, replica-variance check) as the template for future force-field benchmarking on junction motifs. The local OL24 signatures measured here—the sugar-pucker and exchange-point shifts—provide the natural next target: junctions embedded in strained origami lattices, where repeated crossovers could accumulate small local offsets into lattice-scale deformation, should be compared between OL21 and OL24 directly.

## Figures and Tables

**Table 1:** ΔG(open − compact) per force field (bootstrap 95% confidence intervals, CIs, from 16 replicas; 10 ps grid).

Force Field	ΔG Replica-Mean ± s.d.	ΔG Pooled	95% CI
parmbsc1	−0.45 ± 0.20	−0.44	[−0.54, −0.34]
OL21	−0.61 ± 0.16	−0.60	[−0.67, −0.52]
OL24	−0.63 ± 0.27	−0.60	[−0.71, −0.47]

**Table 2:** Simulated vs. crystallographic structural parameters of the junction (crystal values from 1M6G [22] and Section 4.4; simulation values are medians over 16 × 200 ns per force field).

Parameter	1M6G Crystal	parmbsc1	OL21	OL24
IDA (deg, median)	39.3–43.3 (45.4 by our estimator)	51.9	40.9	41.3
Frames with crystal-like IDA (%)	–	2.2	4.6	4.4
Registered WC pairs (median of 20)	20/20	19/20	19/20	19/20
Axial stacking step (Å, median)	≈3.4 (canonical B-DNA)	3.2	3.7	3.4
BII fraction at the junction	0.50	0.226	0.412	0.378
South fraction at the junction	1.00	0.901	0.895	0.818

**Table 3:** Backbone and sugar-pucker substate fractions (per-residue means over all 40 residues, all frames, and 16 replicas per force field; junction residues = the 8 boundary nucleotides; Section 4.4).

Force Field	South (All)	South (Junction)	BII (All)	BII (Junction)
parmbsc1	0.924	0.901	0.227	0.226
OL21	0.906	0.895	0.351	0.412
OL24	0.799	0.818	0.327	0.378

## Data Availability

The data supporting the findings of this study are available within the article and its Supplementary Information Files. The key input files and simulation trajectories are available from the corresponding author upon reasonable request.

## Supplementary Materials

The following supporting information can be downloaded a: https://www.mdpi.com/article/10.3390/ijms27219634/s1.

## Abbreviations

The following abbreviations are used in this manuscript:

Abbreviation	Definition
BI/BII	canonical B-DNA backbone substates, classified by the ε/ζ backbone torsions
CI	confidence interval
CM	Li–Merz divalent-ion parameterization (12-6)
FRET	Förster resonance energy transfer
HJ	Holliday junction
HFE	Li–Merz hydration-free-energy monovalent-ion parameterization
IDA	interduplex angle
OPC	optimal point-charge water model
PDB	Protein Data Bank
PME	particle mesh Ewald
PMF	potential of mean force
SE	standard error
