# Supplementary material for: Molecular Dynamics Simulations Reveal a Broadly Open Solution Ensemble for the DNA Holliday Junction: Mg2+ Crossover Bridging and Weak Force-Field Sensitivity
Source: Int J Mol Sci. 2026 Oct 28;27(21):9634. doi: 10.3390/ijms27219634 (PMC13763174; doi:10.3390/ijms27219634)
Supplement: Supplementary file 1 [file ijms-27-09634-s001.zip › ijms-4622627-supplementary.pdf]

Supporting Information

Molecular Dynamics Simulations Reveal a Broadly Open Solution Ensemble for the DNA Holliday Junction: Mg^{2+} Crossover Bridging and Weak Force-Field Sensitivity

S1. Base-pair registry

The simulation starts from the biological assembly of PDB 1M6G (four copies of d(TCGGTACCGA); Sr^{2+}, waters and hydrogens removed; tleap hydrogen addition). The crystallographic base-pair registry is "sheared": the crossover sits at residues 6|7 of strands A/C but at 4|5 of strands B/D, giving arms of 6, 4, 6 and 4 bp. This is the ≈2.3-Å inter-arm axial offset reported by Thorpe et al. [22].

S2. Dynamic stability metrics

Figure S1 reports per-nucleotide C1′ root-mean-square fluctuations (A), the DNA radius of gyration (B), and the best-fit RMSD to the 1M6G crystal reference (C) for all 48 trajectories (all DNA heavy atoms). The RMSF profile is smallest at the junction-proximal residues (per-force-field minima ≈14–18 Å) and largest at the arm termini (≈22–28 Å); Rg (2.3–2.6 nm) and RMSD (per-replica medians ≈10–28 Å) reach plateaus without upward drift.

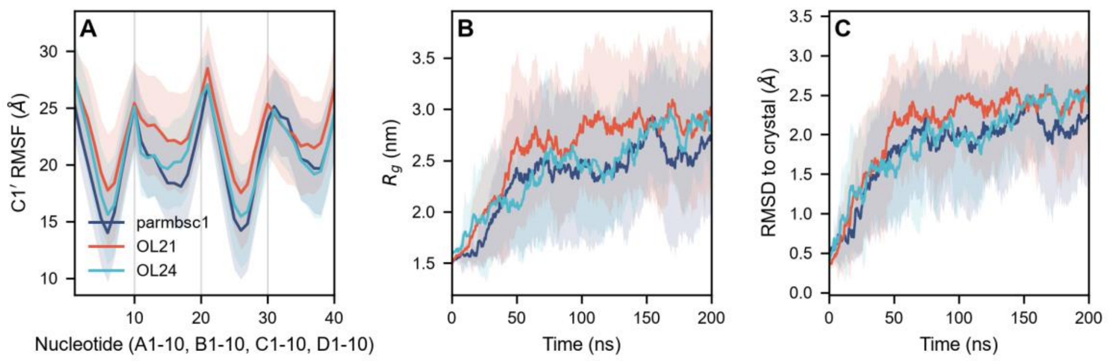

Figure S1. Dynamic stability metrics over all 48 trajectories (mean ± s.d. over the 16 replicas per force field). (**A**) Per-nucleotide C1′ RMSF after superposition onto the crystal reference; vertical grey lines mark the chain boundaries. (**B**) DNA radius of gyration. (**C**) Best-fit RMSD of all DNA heavy atoms to the 1M6G crystal reference. The plateaus without drift in (B) and (C) indicate stable, bounded dynamics in every force field.

S3. Sampling analysis per replica

Table S1 lists, for every replica, the open-state occupancy, Sokal τ_{int} (c = 5), effective sample size, 10-block standard error, the τ_{int}-predicted standard error, and the disjoint-window (20–110 vs. 110–200 ns) occupancy drift.

FF	rep	occupancy (%)	τ_{int} (ns)	N_{eff}	block SE (%)	predicted SE (%)	drift (pp)
parmbsc1	01	73.6	1.31	76	5.73	5.05	17.5
parmbsc1	02	75.0	0.78	128	4.31	3.82	9.1
parmbsc1	03	63.7	1.69	59	6.15	6.26	6.9
parmbsc1	04	70.6	0.87	114	4.24	4.26	13.1
parmbsc1	05	49.8	5.24	19	9.81	11.44	13.4
parmbsc1	06	58.9	1.14	87	5.76	5.26	0.6
parmbsc1	07	64.4	1.38	72	5.83	5.63	18.8
parmbsc1	08	77.3	1.12	89	5.02	4.43	21.6
parmbsc1	09	77.3	0.78	128	4.64	3.70	21.5
parmbsc1	10	62.6	1.10	91	4.13	5.07	5.5
parmbsc1	11	62.4	1.55	65	7.04	6.03	29.2
parmbsc1	12	70.3	0.98	102	5.00	4.53	5.1
parmbsc1	13	71.1	1.07	93	5.58	4.70	25.1
parmbsc1	14	71.8	0.83	120	3.74	4.10	3.6
parmbsc1	15	70.0	1.16	86	5.18	4.94	2.6
parmbsc1	16	63.4	1.05	95	6.33	4.93	2.5
OL21	01	75.7	1.10	91	3.45	4.49	5.9
OL21	02	79.0	0.81	124	3.79	3.65	12.3
OL21	03	72.7	1.86	54	5.78	6.07	20.8
OL21	04	78.7	1.61	62	4.48	5.20	4.3
OL21	05	70.4	0.95	105	4.58	4.46	8.0
OL21	06	73.0	0.80	125	3.76	3.96	14.4
OL21	07	78.7	1.08	92	4.12	4.26	11.1

OL21	08	76.2	1.06	95	5.89	4.38	3.3
OL21	09	61.2	1.07	94	5.55	5.03	10.7
OL21	10	68.5	1.51	66	5.66	5.71	13.2
OL21	11	80.6	1.15	87	4.17	4.24	24.1
OL21	12	71.0	1.43	70	6.65	5.42	18.6
OL21	13	65.2	0.87	115	5.22	4.43	20.6
OL21	14	71.7	1.13	89	5.86	4.79	15.6
OL21	15	74.4	1.67	60	4.90	5.65	12.7
OL21	16	75.9	0.70	142	4.37	3.58	17.5
OL24	01	74.1	1.78	56	5.31	5.85	9.4
OL24	02	73.2	0.82	122	4.65	4.01	12.9
OL24	03	86.6	0.58	173	2.45	2.59	6.4
OL24	04	77.3	2.21	45	6.33	6.22	7.6
OL24	05	70.5	2.14	47	5.87	6.67	2.7
OL24	06	70.8	2.01	50	5.92	6.44	35.4
OL24	07	64.1	3.83	26	7.80	9.39	27.9
OL24	08	74.9	1.31	77	4.93	4.96	19.2
OL24	09	80.8	0.82	122	3.16	3.57	1.7
OL24	10	72.7	0.82	121	5.07	4.05	12.0
OL24	11	56.2	3.53	28	8.74	9.32	21.6
OL24	12	87.1	0.60	167	3.12	2.59	2.8
OL24	13	62.1	1.81	55	6.96	6.53	1.5
OL24	14	67.0	2.05	49	7.21	6.73	19.7
OL24	15	80.5	0.85	118	3.24	3.65	4.0
OL24	16	75.0	1.54	65	5.35	5.37	1.5

S4. The archived assembly-defective system

An earlier build inadvertently simulated two crossing, mutually self-paired duplexes instead of a strand-exchanged junction (a chain-assignment error during construction). Its archived 48 × 200 ns trajectories are re-analysed in Figure S3: the aggregate reproduces the previously reported θ_{23} modes (21°/23°/67°), whereas the experimental 1M6G junction shows none of them (all modes ≈90°, open occupancy 68–73%); its force-field contrasts therefore reflect the defective starting ensemble rather than junction physics.

S5. Per-residue backbone substates

Table S3: South (C2′-endo) and BII fractions per residue, averaged over 16 replicas (✱ = junction-boundary residue; A/C boundary at residue 6|7, B/D at 4|5; – = undefined, the ε/ζ pair requires the next residue and is absent at the 3′-terminal residues). Crystal reference (1M6G coordinates, same definitions): BII fraction 0.50, South 1.00.

**residue**	**parmbsc1 South**	**parmbsc1 BII**	**OL21 South**	**OL21 BII**	**OL24 South**	**OL24 BII**
A1	0.95	0.08	0.92	0.28	0.91	0.31
A2	0.93	0.35	0.93	0.51	0.82	0.51
A3	0.96	0.45	0.94	0.44	0.91	0.33
A4	0.92	0.11	0.92	0.11	0.85	0.09
A5	0.94	0.08	0.88	0.30	0.84	0.38

A6 ✱	0.95	0.18	0.93	0.49	0.91	0.44
A7 ✱	0.96	0.07	0.92	0.83	0.71	0.62
A8	0.91	0.13	0.93	0.22	0.81	0.23
A9	0.95	0.38	0.93	0.44	0.82	0.38
A10	0.91	–	0.85	–	0.59	–
B1	0.95	0.07	0.92	0.25	0.91	0.33
B2	0.91	0.60	0.90	0.61	0.71	0.53
B3	0.95	0.09	0.94	0.19	0.88	0.14
B4 ✱	0.89	0.06	0.88	0.10	0.78	0.08
B5 ✱	0.93	0.66	0.87	0.44	0.89	0.40
B6	0.96	0.18	0.94	0.28	0.90	0.44
B7	0.92	0.17	0.91	0.22	0.68	0.21
B8	0.88	0.26	0.88	0.34	0.67	0.22
B9	0.96	0.25	0.93	0.42	0.82	0.37
B10	0.92	–	0.87	–	0.63	–
C1	0.94	0.10	0.90	0.31	0.90	0.33
C2	0.92	0.49	0.92	0.51	0.82	0.48
C3	0.94	0.29	0.94	0.46	0.90	0.41
C4	0.92	0.07	0.93	0.08	0.85	0.10
C5	0.95	0.06	0.93	0.15	0.93	0.12
C6 ✱	0.96	0.18	0.94	0.55	0.92	0.60
C7 ✱	0.92	0.05	0.85	0.08	0.64	0.08
C8	0.93	0.10	0.89	0.26	0.65	0.17
C9	0.94	0.41	0.92	0.46	0.82	0.36
C10	0.90	–	0.86	–	0.63	–
D1	0.94	0.09	0.91	0.30	0.91	0.35
D2	0.91	0.52	0.93	0.46	0.87	0.42
D3	0.94	0.23	0.93	0.63	0.90	0.50
D4 ✱	0.89	0.15	0.92	0.19	0.83	0.16
D5 ✱	0.72	0.46	0.85	0.60	0.85	0.64
D6	0.94	0.20	0.95	0.25	0.91	0.33
D7	0.91	0.10	0.88	0.16	0.66	0.16
D8	0.89	0.20	0.83	0.32	0.55	0.20
D9	0.95	0.33	0.92	0.40	0.73	0.32
D10	0.91	–	0.86	–	0.60	–

S6. Ion pocket statistics per replica

Table S2: Mean Na^+ count within 4.5 Å of the eight junction phosphates, Mg^{2+} pocket residence fraction, Mg^{2+} unbinding events per trajectory and longest continuous residence.

**FF**	**rep**	**Na^+ (4.5 Å)**	**Mg^{2+} in pocket (%)**	**unbind events**	**longest residence (ns)**
parmbsc1	01	0.74	92.7	253	55
parmbsc1	02	0.74	97.7	116	57
parmbsc1	03	0.79	93.9	283	35
parmbsc1	04	0.92	93.2	269	85
parmbsc1	05	0.84	93.5	220	156
parmbsc1	06	0.77	92.0	319	26
parmbsc1	07	0.88	97.1	54	101
parmbsc1	08	0.73	95.6	143	127
parmbsc1	09	0.93	95.7	195	59

parmbsc1	10	0.83	91.1	238	102
parmbsc1	11	0.74	94.2	210	38
parmbsc1	12	0.63	91.7	359	55
parmbsc1	13	0.80	95.1	226	83
parmbsc1	14	0.67	87.9	441	53
parmbsc1	15	0.99	98.6	58	141
parmbsc1	16	0.74	95.9	132	127
OL21	01	0.71	98.4	58	78
OL21	02	0.71	96.2	157	67
OL21	03	0.67	96.5	139	53
OL21	04	0.59	91.6	319	48
OL21	05	0.63	89.2	391	71
OL21	06	0.52	88.4	533	48
OL21	07	0.62	90.1	430	33
OL21	08	0.84	96.7	225	27
OL21	09	0.68	92.6	350	101
OL21	10	0.64	82.6	494	50
OL21	11	0.68	91.9	295	62
OL21	12	0.93	98.1	101	133
OL21	13	0.65	90.7	392	64
OL21	14	0.82	89.9	402	51
OL21	15	0.66	97.8	76	163
OL21	16	0.78	88.6	323	48
OL24	01	0.63	96.6	125	70
OL24	02	0.59	98.7	54	117
OL24	03	0.57	89.9	370	38
OL24	04	0.69	98.6	54	173
OL24	05	0.86	95.6	226	61
OL24	06	0.66	90.6	320	63
OL24	07	0.65	94.6	211	72
OL24	08	0.99	87.5	379	37
OL24	09	0.53	83.0	586	22
OL24	10	0.70	86.0	477	75
OL24	11	0.63	97.8	125	129
OL24	12	0.59	95.8	269	56
OL24	13	0.76	96.0	185	90
OL24	14	0.70	95.4	140	72
OL24	15	0.64	95.0	204	50
OL24	16	0.60	96.0	131	45

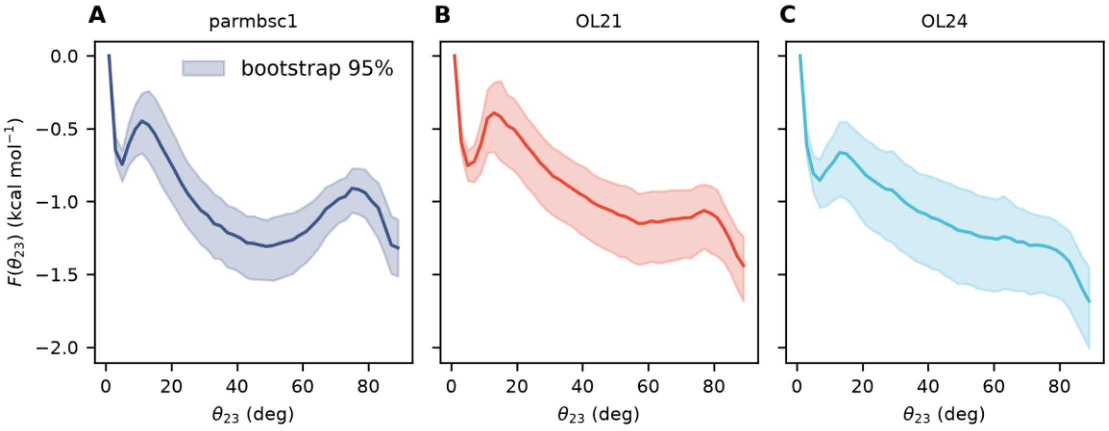

Figure S2. Replica-bootstrap 95% PMF bands.

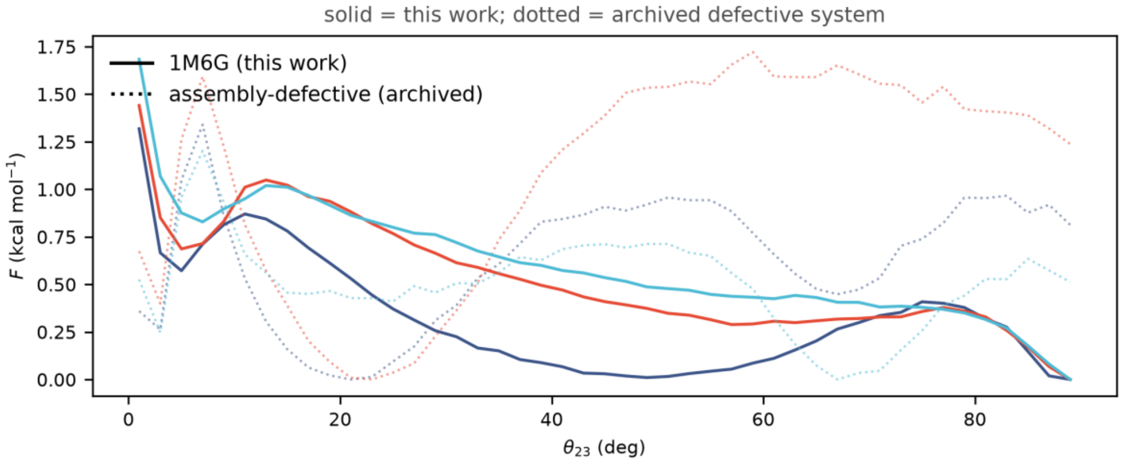

Figure S3. Archived assembly-defective system (dotted) vs. the experimental 1M6G junction (solid). The defective system reproduces the previously reported modes (21°/23°/67°); the experimental junction shows none of them.

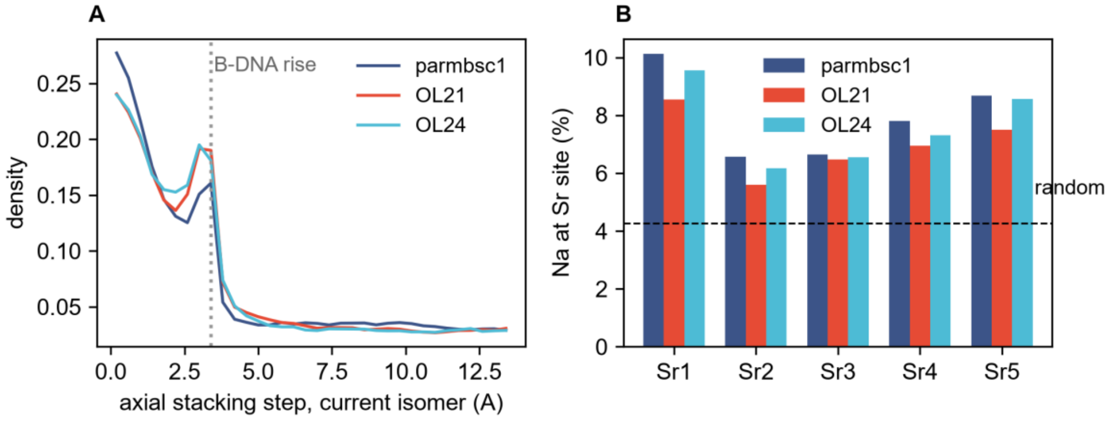

Figure S4. Left: axial stacking-step distributions along the current-isomer registration (canonical B-DNA rise 3.4 Å marked). Right: Na^+ occupancy of the five crystallographic Sr^{2+} sites vs. the random expectation (dashed).

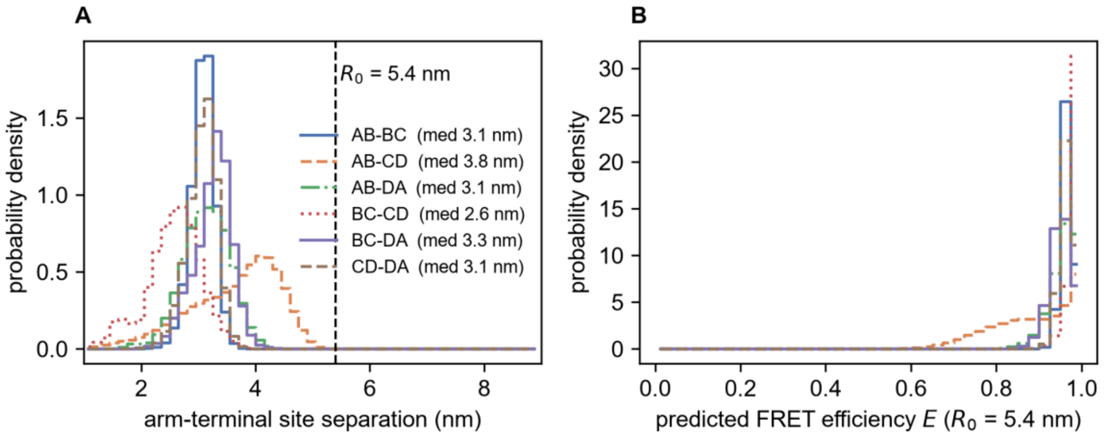

Figure S5. Predicted terminal-dye FRET for the minimal construct. (**A**) Distributions of the six pairwise arm-terminal site separations (C1′–C1′, pooled over all 48 replicas and three force fields); the dashed line marks the Cy3/Cy5 Förster radius R_0 = 5.4 nm, a value typical for this dye pair (cf. ref. [39] of the main text). (**B**) Predicted FRET-efficiency distributions per pair ($E = 1/(1+(R/R_0)^6)$, pooled over force fields). All separations lie below R_0, on the nearly flat shoulder of E(R): predicted efficiencies are uniformly high (per-pair medians 0.89–0.99), i.e., terminal-dye FRET on this 10-mer construct cannot resolve the open ensemble. Resolving the two states instead requires dye separations on the distance-sensitive flank of E(R), as achieved in the classic single-molecule FRET constructs through longer arms and dye positions chosen for distance sensitivity (cf. ref. [3] of the main text).

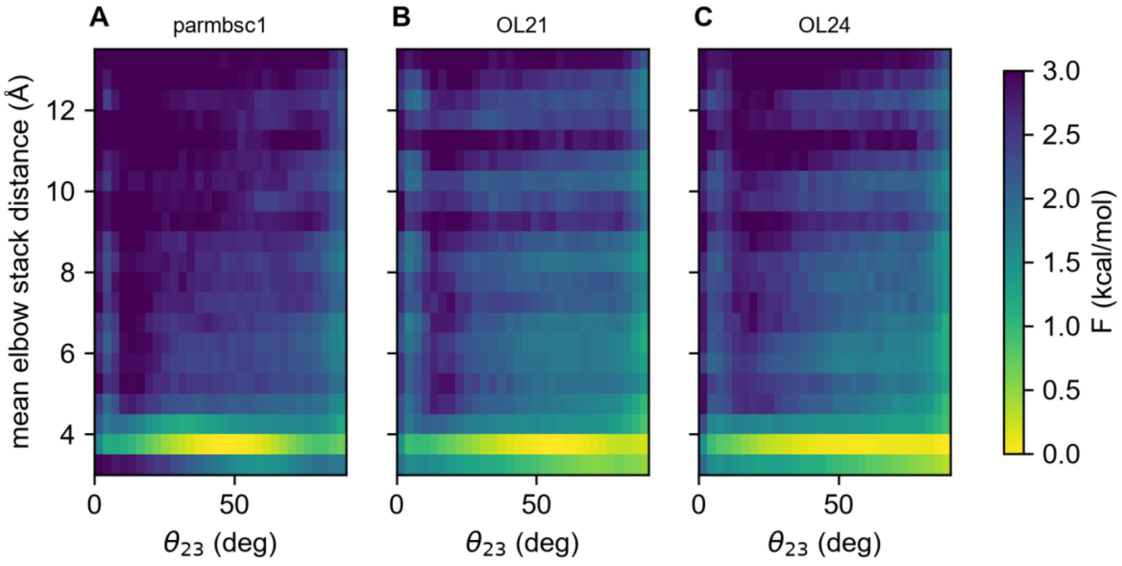

Figure S6. Joint PMF of θ_{23} and the mean elbow packing distance (F < 3 kcal/mol shown). The packing distance remains at its stacked value across the full opening range.

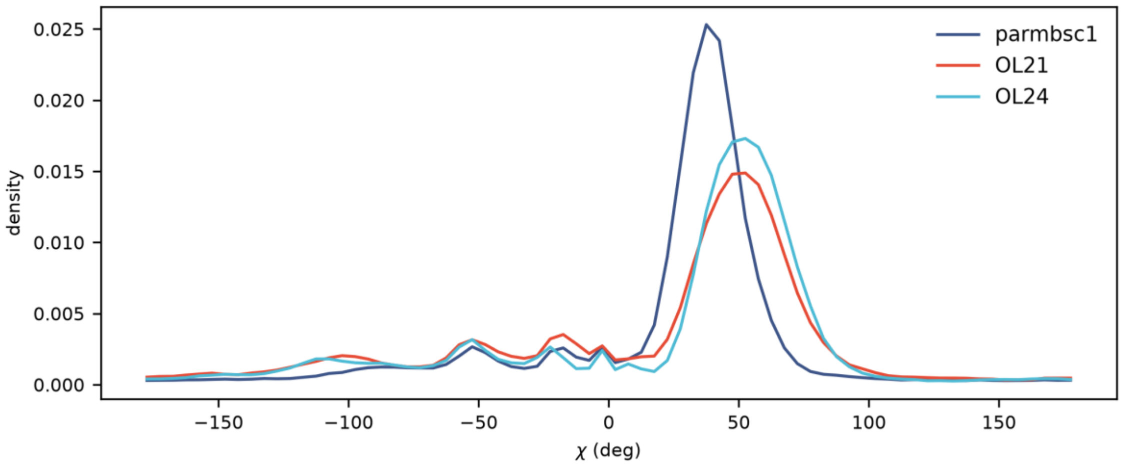

Figure S7. Full circular distributions of the exchange-point pseudodihedral χ.

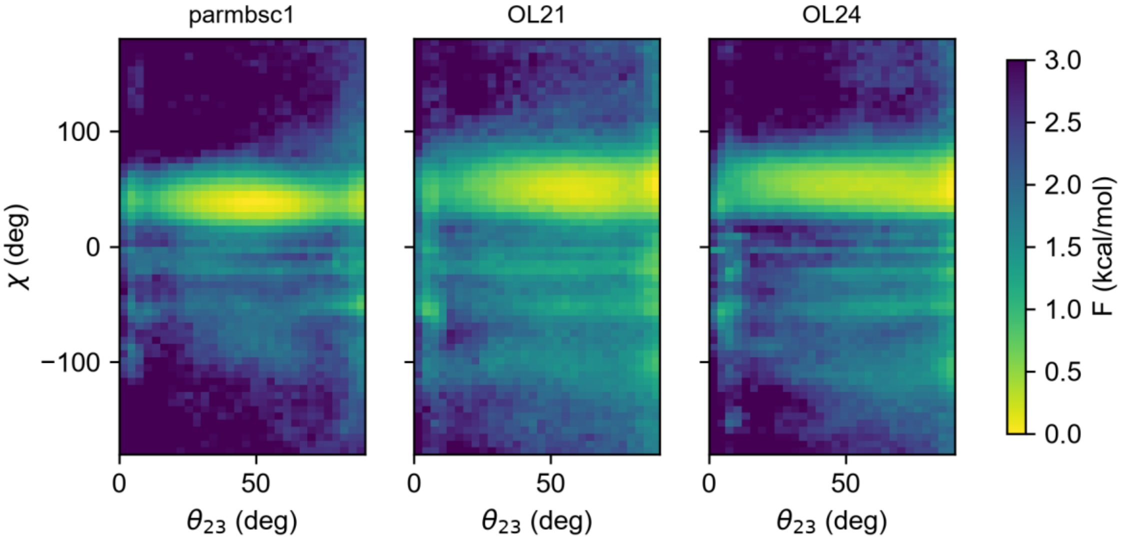

Figure S8. Joint PMF of θ_{23} and χ (F < 3 kcal/mol shown): the two coordinates are near-orthogonal.

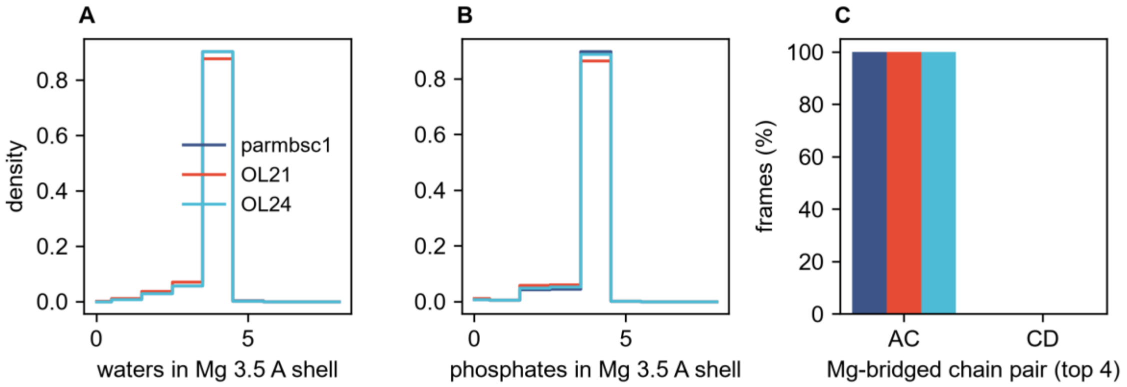

Figure S9. Mg^{2+} coordination. Left: water count in the 3.5-Å shell; middle: phosphate count; right: bridged chain pairs (A/C dominant in all force fields).

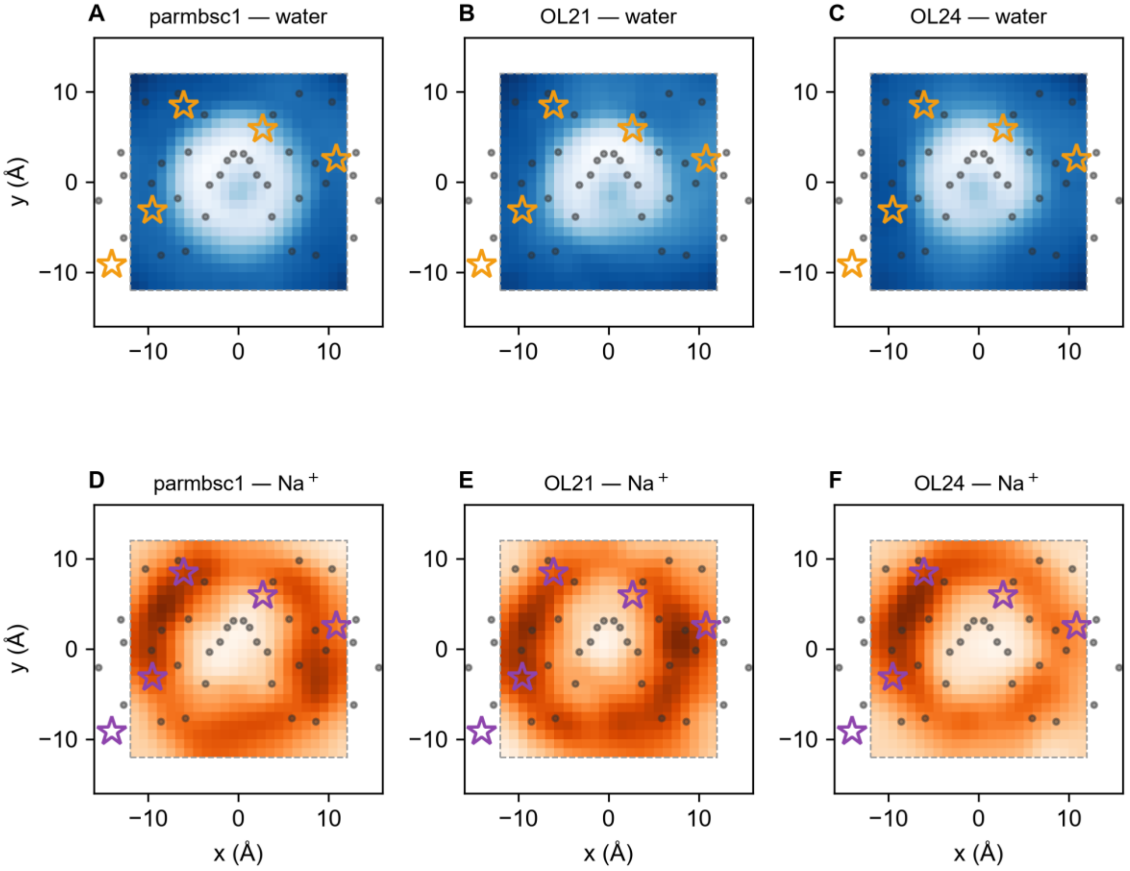

Figure S10. Junction-frame water (top) and Na^+ (bottom) number-density maps (mid-plane slab, $|z| \leq 1.5$ Å), averaged over 16 replicas per force field. Dark dots: projected C1′ atoms of the 1M6G crystal reference (all 40 nucleotides). Stars: the five crystallographic Sr^{2+} positions at their projected (x, y); open stars lie outside the plotted slab.
